# Surgical and clinical determinants of postoperative Hirschsprung‐associated enterocolitis: Multivariate analysis in a large cohort

**DOI:** 10.1002/pdi3.45

**Published:** 2024-05-22

**Authors:** Guoyong Wang, Kai Gao, Rensen Zhang, Qianyang Liu, Cailong Kang, Chunbao Guo

**Affiliations:** ^1^ Department of Pediatric General Surgery Children's Hospital Chongqing Medical University Chongqing China; ^2^ Department of Pediatric General Surgery Women and Children's Hospital, Chongqing Medical University Chongqing China; ^3^ National Clinical Research Center for Child Health and Disorders, Children's Hospital, Chongqing Medical University Chongqing China; ^4^ China International Science and Technology Cooperation Base of Child Development and Critical Disorders, Children's Hospital, Chongqing Medical University Chongqing China; ^5^ Chongqing Key Laboratory of Pediatrics, Children's Hospital, Chongqing Medical University Chongqing China

**Keywords:** evidence level: level 3, Hirschsprung disease, Hirschsprung disease‐associated enterocolitis, intestinal length loss, risk factors, type of HSCR, type of surgery

## Abstract

This research meticulously explores the diverse factors influencing the occurrence of Hirschsprung‐associated enterocolitis (HAEC) subsequent to surgical interventions for congenital megacolon. Considering that Hirschsprung's Disease (HSCR) management predominantly necessitates excision of the aganglionic intestinal segment, the study specifically delineates the correlation between the extent of the excised intestinal segment and the HAEC risk post‐surgery. An analysis of clinical data from 505 patients spanning 2012–2022 enabled a comparison of clinical attributes between patients with and without postoperative HAEC, the application of statistical analyses to identify factors significantly correlating with HAEC, and the determination of independent risk factors via a Logistic regression model. Findings indicate a significant association between preoperative conditions, HSCR variants, and the excised intestinal segment's length with HAEC risk, identifying resection length and albumin levels as independent risk factors. Notably, an increase in resection length by 1 cm correlates with a 9.8% rise in postoperative HAEC risk, whereas a 1 g/L elevation in albumin levels corresponds to a 5.6% risk reduction. Subgroup analyses reaffirm that, across all HSCR variants, an extended resection length significantly elevates HAEC risk. This study underscores the critical roles of albumin levels and the length of the resected intestinal segment as independent risk factors for HAEC post‐congenital megacolon surgery, providing essential insights for clinical strategies aimed at mitigating HAEC risk and enhancing patient care outcomes.

## Graphical abstract

In our study, we found that surgical approach and length of surgical resection significantly impact Hirschsprung‐associated enterocolitis (HAEC) in short‐segment cases, while in long‐segment cases, length of surgical resection and C‐reactive protein level are crucial predictors of HAEC. These findings highlight the importance of tailoring surgical strategies based on disease characteristics.

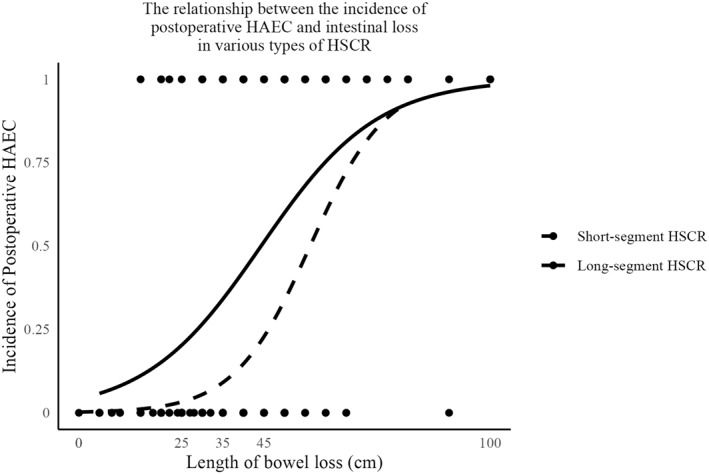

## 1 INTRODUCTION

Hirschsprung's Disease (HSCR) is a common congenital malformation characterized by the absence of ganglion cells in the distal part of the colon or rectum, resulting in functional bowel obstruction and dilation.^1^, ^2^ The disease occurs in approximately 1 in 5000 live births, with males being disproportionately affected. Around 20%–25% of the patients also present with other congenital anomalies or genetic syndromes.^3^, ^4^ HSCR is traditionally classified into short‐segment and long‐segment types based on the extent of the aganglionic zone or the area of the intestine lacking neurons. Hirschsprung's Disease is categorized by the length of the aganglionic segment into five types: short‐segment, common, long‐segment, total colonic, and total intestinal. Short‐segment and common types predominantly involve the rectum and sigmoid colon. In contrast, the long‐segment type affects either the descending or transverse colon. The total colonic type encompasses the ascending colon and the ileum up to 30 cm proximal to the ileocecal junction. The total intestinal type extends to the entire colon and small intestine more than 30 cm from the ileocecal junction, occasionally including the duodenum.^5^, ^6^

Recent advances in understanding the pathophysiology of HSCR have highlighted the role of various gene mutations, such as those affecting the receptor tyrosine kinase and the SOX10 transcription factor, in disrupting the migration of neural crest cells to the distal bowel, leading to the aganglionosis characteristic of HSCR.^7^ The clinical manifestations of HSCR primarily include persistent constipation, abdominal distension, and vomiting, with severe cases potentially leading to life‐threatening enterocolitis. Diagnosis relies on a combination of barium enema, rectal manometry, rectal biopsy, and supportive genetic testing.^8^, ^9^, ^10^ Currently, the most effective treatment for HSCR is surgical resection of the aganglionic intestinal segment, followed by anastomosis of the normal intestinal segment to the anus.^11^, ^12^ Surgical approaches can be categorized into laparoscopic, open, and transanal, each with its own advantages and disadvantages.^13^, ^14^

Despite the effectiveness of surgical treatment, postoperative complications can still occur, including anastomotic leakage, anastomotic stenosis, constipation, diarrhea, and enterocolitis. One particularly severe complication is Hirschsprung‐associated enterocolitis (HAEC), which can lead to diarrhea, abdominal distension, fever, sepsis, and in extreme cases, death.^15^, ^16^ The etiology of HAEC is not fully understood, but it may involve changes in the mucosal barrier, variations in the intestinal microbiota, abnormalities in immune function, and genetic variations.^17^

However, there remains a gap in the literature regarding the impact of various factors on the incidence of HAEC,^18^ especially the relationship between the length of bowel resected during HSCR surgery and the risk of developing HAEC postoperatively. This study aims to retrospectively analyze clinical data from patients who underwent surgery for HSCR at our institution between 2012 and 2022, exploring how HSCR types, surgical approaches, resected bowel length, and other factors affect the incidence of postoperative enterocolitis. We hypothesize that a longer length of bowel removal during surgery may be associated with a higher risk of postoperative HAEC. Through this study, we hope to contribute to the body of knowledge on HSCR and HAEC, improving patient outcomes and informing future research in this field.

## 2 MATERIALS AND METHODS

### 2.1 Patient selection

This study received ethical approval from the institutional review board. It encompassed a cohort of 505 children diagnosed with HSCR who underwent surgical procedures at the Children's Hospital affiliated with Chongqing Medical University within a decade‐long period from January 2012 to December 2022.

The inclusion criteria were (1) meeting the diagnostic criteria for HSCR, confirmed by biopsy to lack ganglion cells, and (2) having undergone surgical treatment with complete surgical records and follow‐up data available.

The exclusion criteria included (1) death due to other congenital malformations or severe complications, (2) incomplete surgical records or missing important data, and (3) loss to follow‐up.

### 2.2 Data collection

A uniformly designed data collection form was used to record patient demographics, clinical characteristics, surgical details, and follow‐up results. The collected data were double‐checked by two trained researchers for accuracy and consistency. The electronic data collection form was stored on a secure network server, accessible and modifiable only by the researchers.

### 2.3 Statistical analysis

Continuous variables were presented as medians and interquartile ranges, and categorical variables as frequencies (percentages). Pearson's χ^2^ and Wilcoxon rank‐sum tests were used for univariate analysis, with a significance level of *p* < 0.05, and all statistical tests were two‐sided. A multivariate logistic regression analysis was conducted, integrating clinically relevant and significant variables derived from the univariate analysis. The length of intestinal loss during surgery was the primary exposure variable, adjusted for the type of HSCR and surgical approach, with postoperative Hirschsprung's‐associated enterocolitis (HAEC) as the primary outcome. A subgroup analysis was performed to evaluate the relationship between different types of HSCR (short‐segment, long‐segment), intestinal tube loss, and postoperative HAEC. All analyses were performed using the R programming language (version 4.2.3).

## 3 RESULTS

**FIGURE 1:**
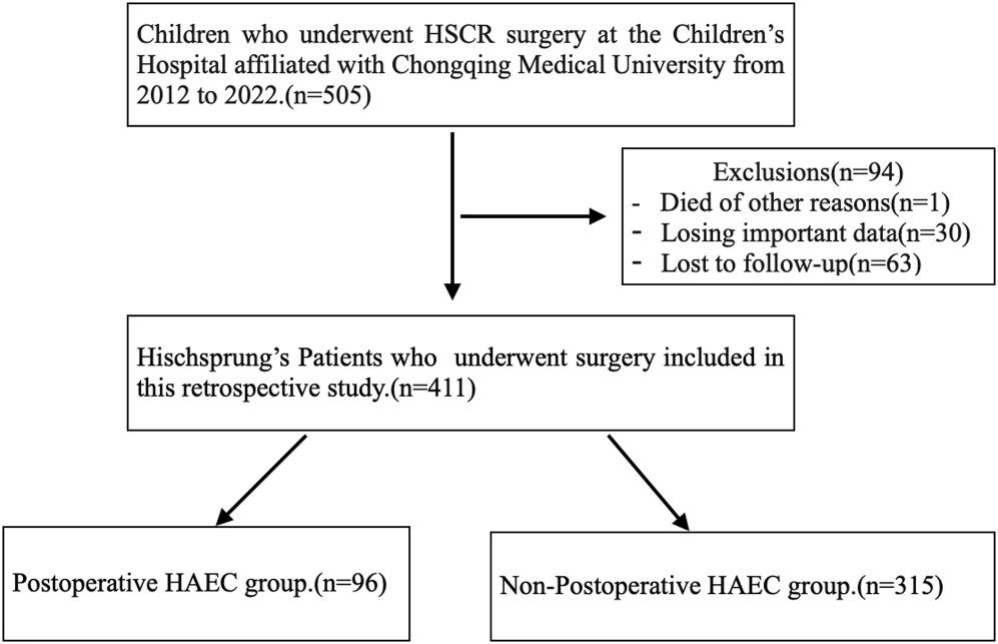
Study flow chart.

The medical records of 505 biopsy confirmed HSCR patients were reviewed. Of these, 94 were excluded: 1 patient died during early follow‐up, 30 patients lacked important data, and 63 failed to follow up, leaving 411 patients for inclusion in the analysis (Figure 1).

The study included 301 cases of short‐segment HSCR, 99 cases of long‐segment, 8 cases of total colon type, and 3 cases that could not be clearly classified due to incomplete clinical data records. The incidence of postoperative HAEC is 10% in the short‐segment group, 11% in the common type, 61% in the long‐segment group, and 30% in the total colonic group. Data on the study cohort of children are shown in Table 1.

**TABLE 1:** Patient characteristics.

Variable	Total (*n* = 411)
Sex	
Male, *n* (%)	351 (85)
Female, *n* (%)	60 (15)
Birth weight (kg), median (IQR)	3.3 [3.1, 3.5]
Age at surgery (months), median (IQR)	7.5 [1.3, 13.5]
Number of hospital admissions, median (IQR)	1 [1, 2]
Other congenital anomalies, *n* (%)	130 (32)
Trisomy 21, *n* (%)	3 (0.7)
Preoperative Hirschsprung‐associated enterocolitis (HAEC), *n* (%)	137 (33)
Preoperative ostomy, *n* (%)	61 (15)
HSCR type, N/A = 5	
Short‐segment, *n* (%)	52 (13)
Common type, *n* (%)	245 (60)
Long‐segment, *n* (%)	99 (24)
Total colonic, *n* (%)	10 (2)
Surgical approach, N/A = 2	
Laparoscopic, *n* (%)	150 (36)
Open surgery, *n* (%)	79 (19)
Transanal, *n* (%)	180 (44)
Postoperative HAEC	
No HAEC, *n* (%)	315 (77)
HAEC, *n* (%)	96 (23)
Laboratory data	
Albumin (g/L), median (IQR)	33.6 [29.7, 37.6]
Hemoglobin (g/L), median (IQR)	99 [90, 109]
C‐reactive protein (CRP) (mg/L)	
<8 mg/L	185 (45)
≥8 mg/L	226 (55)
Procalcitonin (PCT) (ng/L)	
<0.05 ng/L	265 (64.5)
≥0.05 ng/L	146 (35.5)
Length of resection (cm), median (IQR)	35 [25, 45]

*Note*: Continuous variable data does not conform to a normal distribution; it is represented using the median [IQR, interquartile range], while categorical variables are presented as frequencies (%).

Abbreviation: N/A, not available.

### 3.1 Outcome comparison in children

Univariate analysis was conducted to compare patients with postoperative HAEC (*n* = 96) to those without HAEC postoperatively (*n* = 315). For categorical variables such as preoperative ostomy, HSCR type, and surgical method, the Pearson chi‐square (χ^2^) test was utilized to evaluate their association with postoperative HAEC. For continuous variables, such as albumin levels, C‐reactive protein (CRP) levels, and the length of intestinal resection during surgery, which did not follow a normal distribution upon testing, we employedthe Wilcoxon rank‐sum test, a non‐parametric method suitable for data without an assumed distribution.

### 3.2 Child patient baseline characteristics

We compared the differences in a series of baseline characteristics between children who developed HAEC postoperatively (*n* = 96) and those who did not (*n* = 315), conducting a univariate analysis on potential risk factors related to the baseline condition of postoperative HAEC. These factors included gender, other congenital anomalies, presence of HAEC before surgery, birth weight, age at surgery, and number of hospital admissions. The results indicated that none of these factors showed a statistically significant correlation with the risk of postoperative HAEC. Specifically, gender (male vs. female), other congenital anomalies, Trisomy 21 syndrome, and the presence of HAEC before surgery did not demonstrate significant differences statistically (*p*‐values were respectively 0.25, 0.973, 0.783, and 0.387). Numerical variables such as birth weight, age at surgery, and number of hospital admissions also showed no differences when analyzed using the Mann–Whitney *U* test (all *p*‐values were greater than 0.05). These factors reflect the general health condition and potential genetic background of the child patients, but statistically, they did not show a significant association with the occurrence of HAEC postoperatively (see Table 2).

**TABLE 2:** Demographic characteristics of the patients.

Variable type	Factor	Postoperative HAEC (*n* = 96)	No postoperative HAEC (*n* = 315)	X^2^/Mann–Whitney *U* test	*p*‐value
Categorical variables	Sex			1.32	0.25
	Male	78	273		
	Female	18	42		
	Other congenital anomalies	31	99	0.001	0.973
	Trisomy 21	0	3	0.076	0.783
	Preoperative HAEC	28	109	0.749	0.387
Continuous variables	Birth weight (kg)	3.4 [3.1, 3.6]	3.3 [3.1, 3.5]	14,605.0	0.613
	Age at surgery (months)	7.5 [2.2, 11.7]	7.5 [1.2, 16]	15,235.0	0.911
	Number of hospital admissions	1 [1, 2]	1 [1, 2]	15,772.5	0.363

### 3.3 Child Hirschsprung's Disease type and surgical details

We conducted a detailed analysis of the relationship between HSCR type and surgical details and the risk of postoperative HAEC, specifically including factors such as preoperative ostomy, HSCR type, surgical method, and length of surgical resection. The analysis of preoperative ostomy showed a significant increase in the risk of postoperative HAEC for children who had an ostomy (*p* = 0.017), indicating that a preoperative ostomy might be an important predictor of postoperative HAEC risk. Furthermore, the χ^2^ test results for HSCR type were highly significant (*p* < 0.001), revealing significant differences in postoperative HAEC risk among different HSCR subtypes (short‐segment, common, long‐segment, total colonic). Specifically, children with long‐segment HSCR had a significantly higher incidence of postoperative HAEC compared to other types. The analysis of surgical methods also showed significant differences (*p* < 0.001), with laparoscopic surgery, open surgery, and transanal surgery having significantly different associations with the risk of postoperative HAEC, suggesting that different surgical approaches may affect the risk of postoperative HAEC. In terms of numerical variables, the length of surgical resection showed an extremely significant difference (*p* < 0.001) as determined by the Mann–Whitney *U* test, with the median resection length for the postoperative HAEC group being 50 cm (range 40–60 cm), significantly higher than the 30 cm (range 25–40 cm) for the group without postoperative HAEC, indicating that a longer resection length is associated with a higher risk of postoperative HAEC (see Table 3).

**TABLE 3:** Hirschsprung's Disease (HSCR) types and surgical details.

Variable type	Factor	Postoperative HAEC (*n* = 96)	No postoperative HAEC (*n* = 315)	*X* ^2^/Mann–Whitney *U* test	*p*‐value
Categorical variables	Preoperative ostomy	22	39	5.66	0.017
	HSCR type, N/A = 5			103.16	<0.001
	Short‐segment	5	47		
	Common type	27	218		
	Long‐segment	60	39		
	Total colonic	3	7		
	Surgical approach, N/A = 2			53.90	<0.001
	Laparoscopic	64	86		
	Open surgery	17	62		
	Transanal	15	165		
Continuous variables	Length of resection (cm)	50 [40, 60]	30 [25, 40]	4629.00	<0.001

*p < 0.05.

### 3.4 Child laboratory data

We explored the relationship between the occurrence of postoperative HAEC in children and several key laboratory data points, including procalcitonin (PCT), CRP, albumin, and hemoglobin levels. Through the Mann–Whitney *U* test and the χ^2^ test, we evaluated the differences in these indicators between children with and without postoperative HAEC. Initially, for PCT, we categorized the data into less than 0.05 ng/L and greater than or equal to 0.05 ng/L groups, and the results showed no significant association between PCT levels and the occurrence of postoperative HAEC (*χ* ^2^ = 0.4, *p* = 0.526). Similarly, CRP levels were divided into less than 8 mg/L and greater than or equal to 8 mg/L groups, with the results also showing no significant difference (*χ* ^2^ = 2.47, *p* = 0.116), indicating that these two inflammatory markers are not significantly correlated with the risk of postoperative HAEC statistically. However, in terms of numerical variables, albumin levels showed a significant difference between the two groups, with and without postoperative HAEC, with the median for the postoperative HAEC group being 31.9 [28.4, 36.5] g/L, compared to 34.2^19^, ^20^ g/L for the group without HAEC (Mann–Whitney *U* = 17,974.0, *p* = 0.005), suggesting that low albumin levels may be associated with an increased risk of postoperative HAEC. For hemoglobin, although the median for the postoperative HAEC group was slightly lower (98.5 [89, 106.3] g/L compared to 100 [91, 110] g/L), the difference was not statistically significant (Mann–Whitney *U* = 16,655.0, *p* = 0.132) (see Table 4).

**TABLE 4:** Laboratory data for pediatric patients.

Variable type	Laboratory data	Postoperative HAEC (*n* = 96)	No postoperative HAEC (*n* = 315)	Mann–Whitney *U* test/*X* ^2^	*p*‐value
Categorical variables	Procalcitonin (PCT) (ng/L)			0.4	0.526
	<0.05 ng/L	65	200		
	≥0.05 ng/L	31	115		
	C‐reactive protein (CRP) (mg/L)			2.47	0.116
	<8 mg/L	36	149		
	≥8 mg/L	60	166		
Continuous variables	Albumin (g/L)	31.9 [28.4, 36.5]	34.2 [30, 38]	17,974.0	0.005
	Hemoglobin (g/L)	98.5 [89, 106.3]	100 [91, 110]	16,655.0	0.132

*p < 0.05.

### 3.5 Multifactorial logistic analysis of postoperative Hirschsprung‐associated enterocolitis in children

Univariate analysis revealed that the occurrence of postoperative HAEC was significantly associated with preoperative ostomy, hypoalbuminemia, HSCR type, surgical method, and the length of intestinal resection during surgery. Variables significant in univariate analysis were then subjected to multifactorial Logistic regression analysis. The model included resection length (cm), albumin (g/L), surgical approach (laparoscopic, open, and transanal), HSCR type (short‐segment, common, long‐segment, and total colonic), and preoperative ostomy as independent variables. The model results showed that surgical resection length had a significant positive impact on the occurrence of postoperative HAEC (*β* = 0.093, *p* < 0.001), indicating that for each additional 1 cm of resection length, the risk of postoperative HAEC increased by approximately 9.8% (OR = 1.098). Furthermore, for each additional 1 g/L of albumin, the risk of postoperative HAEC decreased by about 5.6% (*β* = −0.058, *p* = 0.044, OR = 0.944). This indicates that surgical resection length and albumin levels are significant predictors of the risk of postoperative HAEC.

In contrast, the impact of the surgical approach, HSCR type, and preoperative ostomy was not as significant as the surgical resection length and albumin levels. Specifically, the surgical approach (laparoscopic, open, and transanal) was not significantly associated with the risk of postoperative HAEC (*p* > 0.05). Similarly, HSCR type (short‐segment, common, long‐segment, and total colonic) and the presence of a preoperative ostomy did not show significant predictive value. We particularly focused on the impact of HSCR type on the risk of postoperative HAEC and set the “common” type as the reference category for the OR value. Compared to the common type, patients with long‐segment HSCR had a significantly increased risk of postoperative HAEC (OR = 8.549, 95% confidence interval (CI): −0.079–4.371, *p* = 0.059), while the risk change for short‐segment and total colonic patients was not significant. This result emphasizes the importance of HSCR type in the risk of postoperative HAEC, especially the need for more attention and management in patients with long‐segment HSCR. In terms of surgical approach, compared to the transanal approach (OR set to 1), the risk change for laparoscopic and open methods was not significant, suggesting that the impact of the surgical approach on postoperative HAEC is not as significant as HSCR type (See Table 5).

**TABLE 5:** Multivariate regression analysis of risk factors for postoperative Hirschsprung‐associated enterocolitis (HAEC).

Factor	*β* (beta)	SE	Wald	*p*‐value	OR (odds ratio)	95% CI lower limit	95% CI upper limit
Constant	−2.877	1.241	−2.319	0.020	0.056	−5.309	−0.446
Preoperative ostomy	−0.498	0.472	−1.055	0.291	0.608	−1.422	0.427
HSCR type							
Common type					1		
Short‐segment	0.302	0.571	−0.529	0.597	0.739	−1.422	0.818
Long‐segment	2.146	1.135	1.890	0.059	8.549	−0.079	4.371
Total colonic	−0.104	1.341	−0.077	0.938	0.901	−2.733	2.525
Surgical approach							
Transanal					1		
Laparoscopic	−0.077	0.441	−0.175	0.861	0.926	−0.941	0.786
Open surgery	−0.741	0.436	−1.699	0.089	0.477	−1.596	0.114
Albumin (g/L)	−0.058	0.029	−2.017	0.044	0.944	−0.114	−0.002
Length of resection (cm)	0.093	0.015	6.304	<0.001	1.098	0.064	0.123

Abbreviation: CI, confidence interval.

*p < 0.05.

### 3.6 Subgroup analysis

Considering the significant impact of different HSCR subtypes and the length of intestinal resection on the incidence of postoperative HAEC, this study conducted a subgroup analysis to explore the association between the length of intestinal damage and the occurrence of postoperative HAEC across different HSCR subtypes. Specifically, the analysis found no significant difference in the incidence of postoperative HAEC between short‐segment and common HSCR types, thus these two subtypes were combined into a short‐segment subgroup for analysis. On the other hand, both total colonic and long‐segment HSCR involve aganglionic segments extending beyond the sigmoid colon. Due to the small sample size of total colonic type, these two subtypes were considered together as a long‐segment subgroup for analysis. This approach aims to more accurately assess the impact of the length of intestinal damage on the risk of postoperative HAEC, taking into account the differences in the length of intestinal resection required by different HSCR subtypes.

**FIGURE 2:**
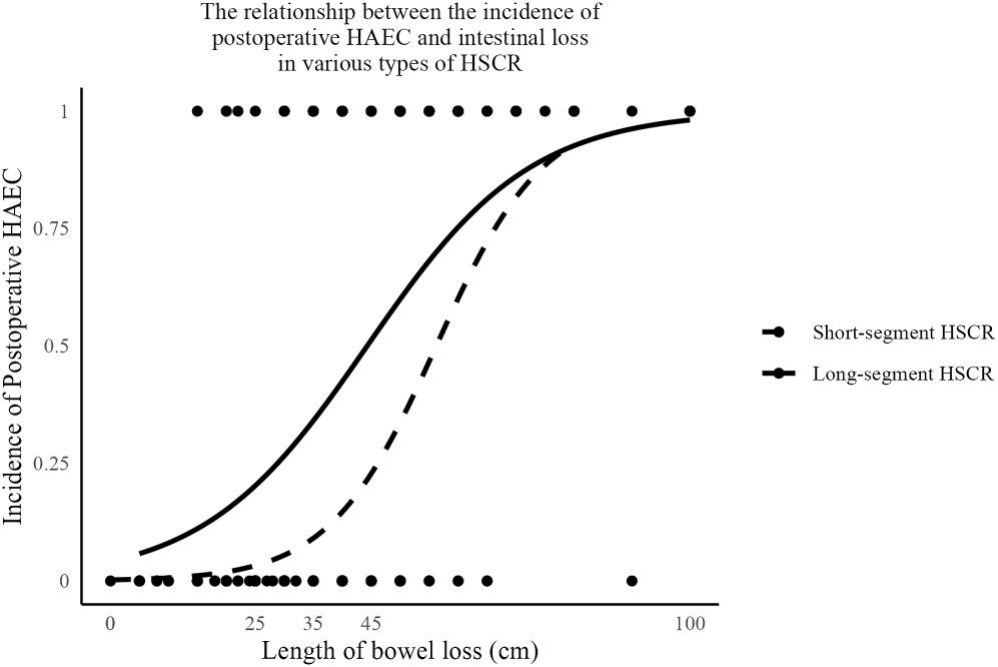
The relationship between the incidence of postoperative Hirschsprung‐associated enterocolitis (HAEC) and intestinal loss in various types of Hirschsprung's Disease (HSCR).

The subgroup analysis showed that, in the short‐segment group, surgical method (*p* = 0.011) and length of resection (*p* < 0.001) significantly affected the occurrence of HAEC, while preoperative ostomy (*p* = 0.797) and albumin levels (*p* = 0.354) were not significantly related to the occurrence of HAEC. In the long‐segment group, the length of surgical resection (*p* < 0.0001) was a significant predictor of HAEC, while preoperative ostomy (*p* = 0.219), surgical method (*p* = 0.764), and albumin levels (*p* = 0.713) were not significant predictors of HAEC. The length of surgical resection significantly affected the occurrence of postoperative HAEC in both subgroups, emphasizing the importance of the length of resection in preventing HAEC. The relationship between the length of resection and the incidence of HAEC is shown in Figure 2.

## 4 DISCUSSION

Hirschsprung‐associated enterocolitis is a major factor affecting mortality and prognosis in patients with HSCR. Our study represents the largest single‐center data sample to date, aimed at identifying risk factors and specifically evaluating the impact of intestinal loss during surgery on postoperative HAEC in various HSCR types. The study shows that the incidence of HAEC varies greatly among published series, ranging from 17% to 50%, with an average incidence of 28.5%.^21^, ^22^ The incidence rate of HAEC in our cohort was 23.4%, consistent with reported data. For total colonic type, previous reports have shown the highest postoperative HAEC incidence rate, reaching 55%.^23^ However, our cohort showed an incidence rate of 30%, which may be due to the smaller number of cases of total colonic type in our study, leading to statistical bias, as a small sample may not accurately reflect the situation in the entire population.

Previous studies have suggested that intestinal loss may lead to the occurrence of postoperative HAEC, but the specific predictive relationship is not clear.^24^ Our study is the first to establish a strong quantitative association between the length of intestinal resection during surgery and the incidence rate of postoperative HAEC. The risk of postoperative HAEC increases with the length of the intestine removed, both in long‐segment and short‐segment HSCR. In short‐segment HSCR, we observed a significant positive correlation between intestinal resection length and HAEC (*p* < 0.001), with each additional centimeter of surgical resection length increasing the likelihood of HAEC by approximately 11.2% (OR = 1.112, 95% CI: 1.066–1.146). Similarly, in long‐segment type, there was a significant positive correlation between surgical resection length and HAEC (*p* < 0.001), with each additional centimeter of surgical resection length increasing the likelihood of HAEC by about 7.4% (OR = 1.074, 95% CI: 1.039–1.109). The correlation between intestinal loss during surgery and HAEC can possibly be explained by the direct impact of this factor on postoperative intestinal functional recovery, metabolism, and microbiota reconstruction. The more intestinal loss, the greater the digestive absorption and immune regulation tasks the remaining intestine must undertake, making it more susceptible to bacterial overgrowth and toxin stimulation.^25^ Therefore, the greater the intestinal loss, the higher the likelihood of HAEC. In the same length of intestinal resection, the increased risk of HAEC is more significant in short‐segment type, possibly due to the different intestinal pathological environments in long‐segment and short‐segment HSCR. Overall, long‐segment HSCR always shows a higher risk of enteritis compared to short‐segment type.^26^

Our findings support previous research that patients with long‐segment HSCR have a higher likelihood of developing postoperative HAEC. For instance, the study by Hagens J et al.^27^ found that longer aganglionic segments might involve a more extensive range of the intestine, leading to longer intestinal resections. In this retrospective cohort study, we observed a significant difference in the occurrence of postoperative HAEC between long‐segment and short‐segment HSCR, with a higher incidence of postoperative HAEC in long‐segment HSCR. This can be attributed to the more extensive intestinal resection in long‐segment HSCR.^28^ Moreover, the extent of surgical resection could not only lead to postoperative HAEC but also affect the overall quality of life and long‐term outcomes of HSCR patients. This emphasizes the necessity of adopting a cautious and personalized approach in surgical treatment, balancing the need to alleviate the disease and the goal of preserving as much healthy intestine as possible to ensure better postoperative recovery and reduce the risk of HAEC.

This study explored a range of potential risk factors brought by basic child patient conditions, including gender, other congenital anomalies, Trisomy 21 syndrome, and the presence of HAEC before surgery, birth weight, age at surgery, and the number of hospital admissions. Our analysis results showed that these factors were not significantly statistically correlated with the risk of postoperative HAEC,^29^, ^30^ indicating that the general health condition and possible genetic background of child patients have no significant statistical association with the occurrence of postoperative HAEC. Contrary to previous reports, earlier studies suggested that female HSCR patients are more likely to develop HAEC postoperatively.^31^ This discrepancy could be attributed to variations in study methodology, patient populations, and other potential confounding factors. Therefore, further research is needed to elucidate this relationship. Preoperative ostomy was identified as a potential risk factor for postoperative HAEC in univariate analysis. However, this association did not remain significant in multivariate analysis, suggesting the influence of other variables, consistent with previous retrospective cohort studies.^19^, ^32^

The surgical method was found to significantly affect the risk of postoperative HAEC, a finding supported by existing literature. Specifically, Demehri et al.^16^ reported that transanal surgery, compared to abdominal surgery, is superior in reducing the risk of postoperative HAEC. This advantage can be attributed to its minimally invasive nature, resulting in less tissue damage and faster postoperative recovery. Furthermore, transanal surgery may minimize the risk of peritoneal adhesions and subsequent intestinal obstruction, a common complication of abdominal surgery. Despite these benefits, some studies have raised concerns about the long‐term efficacy of transanal surgery in restoring colonic nerve function.^33^, ^34^ In our cohort, the risk of HAEC in patients undergoing transanal surgery was reduced by approximately 83.4% (OR = 0.166, 95% CI: 0.092–0.301, *p* < 0.001). However, this association lost its significance in multivariate analysis, suggesting a smaller role of the surgical method in determining the risk of postoperative HAEC. Beltman et al.^35^ reported similar long‐term outcomes in HAEC incidence and functional and quality of life among patients undergoing transanal surgery, conservative treatment, and reoperation. More research is needed to clarify this association and guide the surgical treatment of HSCR patients.

This study included various clinical laboratory markers as potential risk factors for postoperative HAEC. Among them, albumin levels significantly impacted the risk of postoperative HAEC. A retrospective study reported by Du Juan^36^ also indicated that malnutrition is a risk factor for the occurrence of enterocolitis in children with congenital megacolon after surgery. A recent retrospective study^37^ identified microcytic hypochromic anemia as a risk factor for postoperative HAEC, but our cohort study did not find a significant association between hemoglobin levels and postoperative HAEC. In this study, other markers such as CRP, PCT, and hemoglobin did not show a significant association in regression analysis, consistent with the conclusions of our study. The relationship between CRP, PCT, hemoglobin, and postoperative HAEC remains controversial, requiring more research to clarify their roles.^20^, ^38^

Previous studies from various medical centers have reported differences in the incidence of postoperative HAEC, which could be attributed to different diagnostic criteria, surgical techniques, and management strategies.^39^, ^40^ In our analysis of surgical data, we discovered the relationship between the length of intestinal loss and postoperative HAEC, further clarifying that preserving as much of the normal intestine as possible during surgery significantly reduces the risk of enterocolitis after surgery for all types of megacolon. However, to confirm whether the minimizing resection while preserving sufficient intestine leads to additional surgery or recurrence of HSCR,^41^ more prospective studies and randomized controlled trials involving more medical centers are needed in the future.

This study does indeed have certain limitations, mainly due to its observational design and challenges in data collection. Firstly, the data for this study were collected from the pediatric general surgery department of our hospital, a single, independent center, and the process of data collection could inevitably be influenced by the researchers’ habits, clinical experience, and overall understanding of the disease. We hope that in the future, it will be possible to collaborate with multiple centers for data collection and have different researchers independently perform statistical analysis of the research data to obtain better research results. Secondly, we were unable to verify the effectiveness of probiotics as a protective factor in preventing HAEC. The routine use of probiotics postoperatively, along with additional independent use by patients’ families, blurs their direct impact on preventing HAEC.^11^, ^42^ Thirdly, our institution's diagnostic criteria for HAEC are mainly based on clinical data and follow‐up, which are more lenient compared to the scoring criteria used in prospective studies,^43^ potentially resulting in a relatively higher incidence of HAEC reported in our study. Using more standardized diagnostic methods could produce more precise outcomes. Additionally, the average age of HAEC patients in our study was higher than in previous studies. This could be attributed to our study population including patients of all age groups. Despite these limitations, our study provides valuable data on risk factors for postoperative HAEC. To address these limitations, future research needs to enhance our understanding of the various factors influencing the risk of postoperative HAEC. Moreover, adopting more standardized diagnostic criteria and including a larger and more diverse study population may help better understand the risks and potential protective factors of HAEC.

## 5 CONCLUSION

In summary, this study highlights the roles of albumin levels and intestinal length loss as independent risk factors for postoperative HAEC. The findings suggest that, across all types of HSCR, the greater the loss of intestinal length, the higher the risk of postoperative HAEC. These findings provide strong evidence of the importance of preserving normal intestinal length during surgery. Additionally, they offer new insights and strategies for preventing postoperative HAEC, thereby improving patient outcomes.

## 6 RECOMMENDATIONS

Based on the findings of this study, we strongly recommend minimizing the loss of intestinal length during surgery in children with HSCR to reduce the risk of HAEC. At the same time, closely monitoring postoperative intestinal function and microbial status to quickly identify and address any signs of HAEC is crucial. Furthermore, we advocate for more multicenter, large‐scale, prospective studies to confirm the findings of this study and explore other potential factors and mechanisms that may influence the incidence of HAEC.

## AUTHOR CONTRIBUTIONS

**Guoyong Wang**: Conceptualization (lead); Data Curation (lead); Formal Analysis (lead); Writing – Original Draft Preparation (lead); Writing – Review & Editing (lead). **Kai Gao**: Conceptualization (lead); Formal Analysis (supporting). **Cailong Kang**: Writing – Review & Editing (lead). **Rensen Zhang**: Writing – Review & Editing (lead). **Qianyang Liu**: Data Curation (supporting); Formal Analysis (supporting). **Chunbao Guo**: Writing – Original Draft Preparation (lead). All authors have read and approved the final manuscript as submitted and agree to be accountable for all aspects of the work.

## CONFLICT OF INTEREST STATEMENT

The authors declare that they have no competing interests.

## ETHICS STATEMENT

The study involves the use of human participants’ medical records. All procedures performed in this study were approved by the Medical Research Ethics Committee of the Affiliated Children's Hospital of Chongqing Medical University.

## INFORMED CONSENT

The study was granted an exemption from requiring informed consent for the following reasons: The research utilized previously collected clinical diagnostic and treatment records/biological samples. The risk to participants' privacy was deemed significant as informed consent documents would be the only linkage to the research, with the primary risk being the potential disclosure of their identity or personal information. The research posed minimal risk to participants, akin to procedures and actions outside of a research setting that do not require written informed consent, such as interviews or telephone/email surveys.

## Data Availability

The data that support the findings of this study are available on request from the corresponding author. The data are not publicly available due to privacy or ethical restrictions.

## References

[1] Yan BL , Bi LW , Yang QY , Wu XS , Cui HL . Transanal endorectal pull‐through procedure versus transabdominal surgery for Hirschsprung disease: a systematic review and meta‐analysis. Medicine. 2019;98(32):e16777. 31393401 10.1097/MD.0000000000016777 PMC6709203

[2] Muehsam E . Modern concept of congenital megacolon (Hirschsprung's disease). Am J Dig Dis. 1946;13(1):3‐9. 21010982 10.1007/BF03002744

[3] Liu CP , Tang QQ , Lou JT , et al. Association analysis of the RET proto‐oncogene with Hirschsprung disease in the Han Chinese population of southeastern China. Biochem Genet. 2010;48(5‐6):496‐503. 20454948 10.1007/s10528-010-9333-4

[4] Yang D , Yang J , Li S , et al. Effects of RET, NRG1 and NRG3 polymorphisms in a Chinese population with Hirschsprung disease. Sci Rep. 2017;7(1):43222. 28256518 10.1038/srep43222 PMC5335705

[5] Tam PKH , Garcia‐Barceló M . Genetic basis of Hirschsprung’s disease. Pediatr Surg Int. 2009;25(7):543‐558. 19521704 10.1007/s00383-009-2402-2

[6] Stewart DR , von Allmen D . The genetics of Hirschsprung disease. Gastroenterol Clin N Am. 2003;32(3):819‐837. 10.1016/s0889-8553(03)00051-7 14562576

[7] Heanue TA , Pachnis V . Enteric nervous system development and Hirschsprung's disease: advances in genetic and stem cell studies. Nat Rev Neurosci. 2007;8(6):466‐479. 17514199 10.1038/nrn2137

[8] Kenny SE , Tam PK , Garcia‐Barcelo M . Hirschsprung's disease. Semin Pediatr Surg. 2010;19(3):194‐200. 20610192 10.1053/j.sempedsurg.2010.03.004

[9] Ma Y , Jiang Q , Zhang Z , et al. Diagnosis of Hirschsprung disease by hydrocolonic sonography in children. Eur Radiol. 2022;32(3):2089‐2098. 34532759 10.1007/s00330-021-08287-w

[10] Xiao J , Hao LW , Wang J , et al. Comprehensive characterization of the genetic landscape of familial Hirschsprung's disease. World J Pediatr. 2023;19(7):644‐651. 36857021 10.1007/s12519-023-00686-x PMC10258170

[11] Hersh J . Congenital megacolon Hirschsprung's disease; megacolon treated by segmental resection. Am J Surg. 1947;74(6):815‐819. 20271722 10.1016/0002-9610(47)90400-5

[12] Jensen AR , Frischer JS . Surgical history of Hirschsprung disease. Semin Pediatr Surg. 2022;31(2):151174. 35690466 10.1016/j.sempedsurg.2022.151174

[13] Demehri FR , Dickie BH . Reoperative techniques and management in Hirschsprung disease: a narrative review. Transl Gastroenterol Hepatol. 2021;6:42. 34423163 10.21037/tgh-20-224 PMC8343417

[14] Skarsgard ED , Superina RA , Shandling B , Wesson DE . Initial experience with one‐stage endorectal pull‐through procedures for Hirschsprung's disease. Pediatr Surg Int. 1996;11(7):480‐482. 24057788 10.1007/BF00180088

[15] Frykman PK , Kim S , Wester T , et al. Critical evaluation of the Hirschsprung‐associated enterocolitis (HAEC) score: a multicenter study of 116 children with Hirschsprung disease. J Pediatr Surg. 2018;53(4):708‐717. 28760457 10.1016/j.jpedsurg.2017.07.009 PMC6247908

[16] Zhu T , Zhang G , Meng X , et al. Enterocolitis is a risk factor for bowel perforation in neonates with Hirschsprung's disease: a retrospective multicenter study. Front Pediatr. 2022;10:807607. 35198516 10.3389/fped.2022.807607 PMC8859433

[17] Jiao CL , Chen XY , Feng JX . Novel insights into the pathogenesis of Hirschsprung's‐associated enterocolitis. Chin Med J. 2016;129(12):1491‐1497. 27270548 10.4103/0366-6999.183433 PMC4910376

[18] Le‐Nguyen A , Righini‐Grunder F , Piché N , Faure C , Aspirot A . Factors influencing the incidence of Hirschsprung associated enterocolitis (HAEC). J Pediatr Surg. 2019;54(5):959‐963. 30808539 10.1016/j.jpedsurg.2019.01.026

[19] Peng C , Chen Y , Zhang T , et al. Treatment and outcome of early symptomatic anastomotic leakage after surgery for congenital megacolon. Chin J Pediatri Surg. 2018;39(12):895‐899.

[20] Wall N , Kastenberg Z , Zobell S , Mammen L , Rollins MD . Use of an enterocolitis triage and treatment protocol in children with Hirschsprung disease reduces hospital admissions. J Pediatr Surg. 2020;55(11):2371‐2374. 32553451 10.1016/j.jpedsurg.2020.05.004

[21] Huang W , Li X , Zhang J , Wang W , Zhang S . Incidence and risk factors of early complications after surgery for congenital megacolon. J Clin Pediatr Surg. 2018;17(02):99‐105.

[22] Austin KM . The pathogenesis of Hirschsprung’s disease‐associated enterocolitis. Semin Pediatr Surg. 2012;21(4):319‐327. 22985837 10.1053/j.sempedsurg.2012.07.006

[23] Wildhaber BE , Teitelbaum DH , Coran AG . Total colonic Hirschsprung's disease: a 28‐year experience. J Pediatr Surg. 2005;40(1):203‐207. 15868586 10.1016/j.jpedsurg.2004.09.033

[24] Zeng J , Xu X . Rethinking the standardization of diagnosis and treatment for congenital megacolon. J Clin Pediatr Surg. 2021;20(03):201‐207.

[25] Hackam DJ , Filler RM , Pearl RH . Enterocolitis after the surgical treatment of Hirschsprung's disease: risk factors and financial impact. J Pediatr Surg. 1998;33(6):830‐833. 9660207 10.1016/s0022-3468(98)90652-2

[26] Cheng S , Wang J , Pan W , et al. Pathologically assessed grade of Hirschsprung‐associated enterocolitis in resected colon in children with Hirschsprung's disease predicts postoperative bowel function. J Pediatr Surg. 2017;52(11):1776‐1781. 28385428 10.1016/j.jpedsurg.2017.03.056

[27] Hagens J , Reinshagen K , Tomuschat C . Prevalence of Hirschsprung‐associated enterocolitis in patients with Hirschsprung disease. Pediatr Surg Int. 2022;38(1):3‐24. 34595554 10.1007/s00383-021-05020-y PMC8732830

[28] Sakurai T , Tanaka H , Endo N . Predictive factors for the development of postoperative Hirschsprung‐associated enterocolitis in children operated during infancy. Pediatr Surg Int. 2021;37(2):275‐280. 33245447 10.1007/s00383-020-04784-z

[29] Kwendakwema N , Al‐Dulaimi R , Presson AP , et al. Enterocolitis and bowel function in children with Hirschsprung disease and trisomy 21. J Pediatr Surg. 2016;51(12):2001‐2004. 27670962 10.1016/j.jpedsurg.2016.09.026

[30] Chantakhow S , Tepmalai K , Singhavejsakul J , Tantraworasin A , Khorana J . Prognostic factors of postoperative Hirschsprung‐associated enterocolitis: a cohort study. Pediatr Surg Int. 2023;39(1):77. 36622463 10.1007/s00383-023-05364-7

[31] Demehri FR , Halaweish IF , Coran AG , Teitelbaum DH . Hirschsprung‐associated enterocolitis: pathogenesis, treatment and prevention. Pediatr Surg Int. 2013;29(9):873‐881. 23913261 10.1007/s00383-013-3353-1

[32] Zhang J , Ma T , Peng Y , Huang G , Liu F . A 5‐year follow‐up study of neonates with Hirschsprung's disease undergoing transanal Soave or Swenson surgery. Patient Prefer Adherence. 2017;11:1957‐1961. 29238171 10.2147/PPA.S149722 PMC5716314

[33] Mao YZ , Tang ST , Li S . Duhamel operation vs. transanal endorectal pull‐through procedure for Hirschsprung disease: a systematic review and meta‐analysis. J Pediatr Surg. 2018;53(9):1710‐1715. 29137805 10.1016/j.jpedsurg.2017.10.047

[34] Beltman L , Labib H , Ahmed H , et al. Transition zone pull‐through in patients with Hirschsprung disease: is redo surgery beneficial for the long‐term outcomes? J Pediatr Surg. 2023;58(10):1903‐1909. 36941171 10.1016/j.jpedsurg.2023.02.043

[35] Du J . Risk factors and preventive measures for postoperative enterocolitis in children with congenital megacolon. Nurs Pract Res, 2021;18(01):68‐71.

[36] Huang Y , Ren H . Microcytic hypochromic Anemia is a risk factor for postoperative HAEC: a retrospective study. Front Surg. 2023;10:1055128. 36874458 10.3389/fsurg.2023.1055128 PMC9975337

[37] Gunadi , Luzman RA , Kencana SMS , et al. Comparison of two different cut‐off values of scoring system for diagnosis of Hirschsprung‐associated enterocolitis after transanal endorectal pull‐through. Front Pediatr. 2021;9:705663. 34485196 10.3389/fped.2021.705663 PMC8415414

[38] Umeda S , Kawahara H , Yoneda A , et al. Impact of cow's milk allergy on enterocolitis associated with Hirschsprung's disease. Pediatr Surg Int. 2013;29(11):1159‐1163. 23982385 10.1007/s00383-013-3379-4

[39] Svetanoff WJ , Lopez JJ , Briggs KB , et al. Management of Hirschsprung associated enterocolitis‐How different are practice strategies? An international pediatric endosurgery group (IPEG) survey. J Pediatr Surg. 2022;57(6):1119‐1126. 35282932 10.1016/j.jpedsurg.2022.01.036

[40] Zeng J . Issues related to Unplanned reoperation for congenital megacolon. J Clin Pediatr Surg. 2018;17(02):94‐98.

[41] Nakamura H , Lim T , Puri P . Probiotics for the prevention of Hirschsprung‐associated enterocolitis: a systematic review and meta‐analysis. Pediatr Surg Int. 2018;34(2):189‐193. 28983778 10.1007/s00383-017-4188-y

[42] Pastor AC , Osman F , Teitelbaum DH , Caty MG , Langer JC . Development of a standardized definition for Hirschsprung's‐associated enterocolitis: a Delphi analysis. J Pediatr Surg. 2009;44(1):251‐256. 19159752 10.1016/j.jpedsurg.2008.10.052

[43] Gunadi , Sukarelawanto AVR , Ritana A , et al. Postoperative enterocolitis assessment using two different cut‐off values in the HAEC score in Hirschsprung patients undergoing Duhamel and Soave pull‐through. BMC Pediatr. 2020;20(1):457. 33008355 10.1186/s12887-020-02360-x PMC7531158

